# Supplementary material for: Comparative multiomics analysis of cell physiological state after culture in a basket bioreactor
Source: Sci Rep. 2022 Nov 23;12:20161. doi: 10.1038/s41598-022-24687-4 (PMC9686226; doi:10.1038/s41598-022-24687-4)
Supplement: Supplementary file 1 — Supplementary Information 1. [file 41598_2022_24687_MOESM1_ESM.zip › raw data/Transcriptomic raw data/6.Enrichment/1.GO/TE1vsTC1/DOWN/TE1vsTC1.down_GObar_bp.pdf]

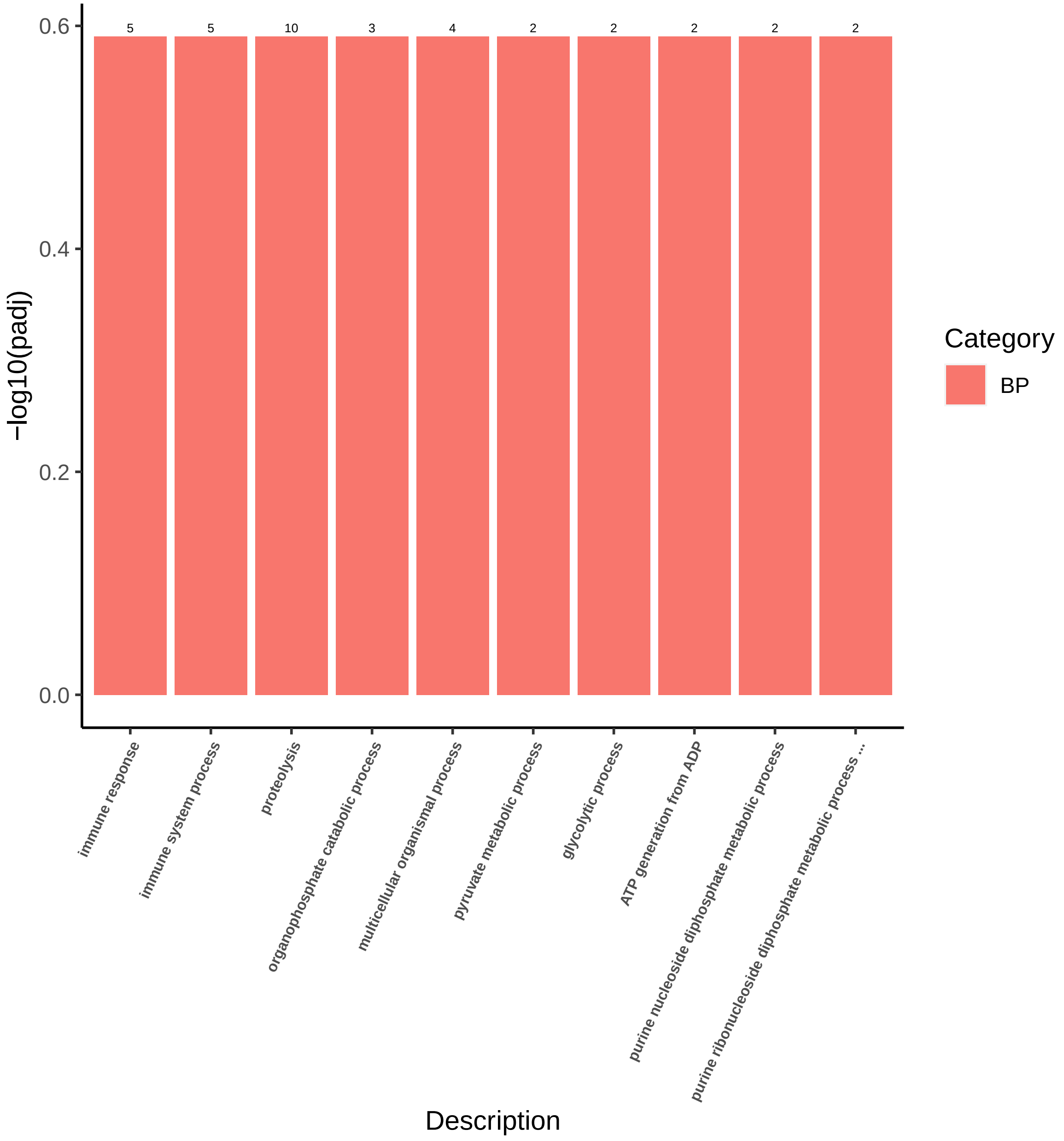

0.6
0.4
0.2
0.0
−log10(padj)
5
5
10
3
4
2
2
2
2
2
immune response
immune system process
proteolysis
organophosphate catabolic process
multicellular organismal process
pyruvate metabolic process
glycolytic process
ATP generation from ADP
purine nucleoside diphosphate metabolic process
purine ribonucleoside diphosphate metabolic process ...
Description
Category
BP
